# Bacterial Spectrum, Isolation Sites and Susceptibility Patterns of Pathogens in Adult Febrile Neutropenic Cancer Patients at a Specialist Hospital in Saudi Arabia

**DOI:** 10.14740/wjon850w

**Published:** 2014-12-03

**Authors:** Mansoor Sirkhazi, Azmi Sarriff, Noorizan Abd Aziz, Fatma Almana, Osama Arafat, Mahmoud Shorman

**Affiliations:** a Department of Pharmacy, King Fahad Specialist Hospital, Dammam, Saudi Arabia; b School of Pharmaceutical Sciences, University Sains Malaysia, 11800 Minden, Penang, Malaysia; c Faculty of Pharmacy, University Technologia MARA, Selengor DE, Malaysia; d Infection Control Department, King Fahad Specialist Hospital, Dammam, Saudi Arabia; e Internal Medicine Department, Marshall University, Huntington, WV 25701, USA

**Keywords:** Febrile neutropenia, Bacterial isolates, Cancer type and isolation sites

## Abstract

**Background:**

Knowing local spectrum and sensitivity for bacterial isolates causing febrile neutropenia is important as starting an appropriate empirical antibiotic therapy is considered a medical emergency in these high-risk patients.

**Methods:**

A retrospective study of a total of 106 microbiologically febrile episodes in hospitalized adult neutropenic cancer patients, who were admitted from May 2009 to May 2013, at King Fahad Specialist Hospital, Dammam, Saudi Arabia, was conducted.

**Results:**

Among 106 microbiologically documented febrile neutropenic episodes, the majority of malignancies were solid tumors accounting for 53.8% (57/106) and hematological malignancies accounted for 46.23% (49/106). The most common malignancies were non-Hodgkin’s lymphoma 19.81% (21/106) followed by acute myeloid leukemia 15.09% (16/106), then colorectal cancer 13.21% (14/106), pancreatic cancer and acute lymphoblastic leukemia accounting for 5.66% (6/106) each, multiple myeloma 4.72% (5/106), gall bladder cancer 3.77% (4/106), and lung cancer 2.83% (3/106). A total of 138 bacterial isolates were identified. The overall prevalence of gram-negative bacteria was 65.94% (91/138) and for gram-positive bacteria was 34.06% (47/138). The most common bacterial isolation sites were blood 33.32% (46 isolates), urine 29.71% (41 isolates), wound 19.55% (27 isolates), body fluids 9.41% (13 isolates) and sputum 7.96% (11 isolates). The most predominant pathogens were *Escherichia coli* 30.43 (42/138), *Klebsiella pneumonia* 14.49% (20/138), *Staphylococcus aureus* 13.04% (18/138), *Sptreptococcus* spp. 7.25% (10/138), *Pseudomonas* spp. 7.25% (10/138), *Enterococcus* spp. 5.80% (8/138), *Staphylococcus* spp. 4.35% (6/138), *Corynebacterium* spp. 3.62% (5/138), *Enterobacter* spp. 3.62% (5/138), *Acinobacter* spp. 2.90% (4/138), *Serratia marcescens* 2.17% (3/138), *Proteus mirabilis* 1.45% (2). *Aeromonas hydrophylia, Citrobacter freundii, Providencia stuartii, Sphingomonas paucimobilis* and *Stenotropomonas multipholia* contributed to 0.72% with one isolate each. For gram-negative *Escherichia coli* and *Klebsiella pneumonia*, the extended-spectrum beta-lactamases producers (ESBLs) rates were 38% and 22.22% respectively. For Pseudomonas aerugenosa imipenem-cilastatin resistance rate was 18.84%. For gram-positive bacteria, methicillin-resistant *Staphylococcus aureus* (MRSA) rate was 28.62%. The vancomycin-resistant *Enterococci* (VRE) rate was 1.18%.

**Conclusion:**

Gram-negative bacteria were more prevalent as a cause of infection in adult cancer patients with febrile neutropenia at our institution, with *Escherichia coli* and *Klebsiella pneumonia* with high ESBLs rates being the most common pathogens. Blood stream infections followed by urinary tract infections were the most common sites of infection. The use of initial antibiotic therapy in febrile neutropenic episodes should be based on local bacterial spectrum and susceptibility/sensitivity patterns to prevent treatment failure with increased morbidity and mortality.

## Introduction

Febrile neutropenia is an adverse effect and serious complications of cytotoxic chemotherapy treatment in cancer patients. Oral temperature ≥ 38.3 °C or ≥ 38 °C for more than 1 h, when absolute neutrophill count < 500 cells/mm^3^ or a count of < 1,000 cells/mm^3^ with predicted decrease to < 500 cells/mm^3^, are considered as an increased risk of infection and mortality. Fever may be the only manifestation of underlying infections following chemotherapy treatment in cancer patients [1, 2]. Specific causative pathogens are identified only in 20-25% of febrile neutropenic patients [3].

Infection remains the principal complication in netropenic cancer patients. During 1960s and 1970s, 80% of mortality in hematological malignancies was due to infection [4]. Nowadays, the mortality rate is reduced to 20%. The source of bacterial infection in neutropenic patients varies according to the severity and duration of neutropenia [5]. The most common site of infections in neutropenic patients is respiratory tract infections followed by blood stream, urinary tract, skin and soft tissues, oro-pharynx and gastrointestinal tract [4].

In the 1970s gram-negative pathogens were prevailing later in 1980s and 1990s the gram-positive bacteria were emerging [2]. The International American Therapy Cooperative Group (IATCG) of the European Organization for Research and Cancer Treatment of Cancer (EORTC) reported that gram-positive pathogens were dominant from 1973 to 1994 and later that same trial observed that the rate of gram-negative bacteriemias dropped from 71% to 31% [6]. Randomized controlled trials conducted by other centers observed the same shifting pattern of the bacterial spectrum in neutropenic cancer patients [7]. The reason for this change in the etiology of pathogens remains unclear [8]. Several studies assumed that the shift of infecting pathogens towards more gram-positive was due to long-term indwelling catheters, aggressive chemotherapy, continuous evolution of antibiotic use and changes in clinical and local antibiotic resistance [9, 10].

Recently the etiology of infecting pathogens changed again. Several studies from United States and Europe reported the re-emergence of gram-negative bacteria in neutropenic cancer patients [9, 11].

The antimicrobial therapy must be initiated as soon as the infection is suspected in febrile neutropenic patients [4]. Older reports showed that if early empirical antibiotic therapy is not administered, due to gram-negative bacteria in severe neutropenic patients, the mortality rate reached up to 40%. Although later reports showed lower mortality reports but the high mortality justified the initiation of early broad spectrum antibiotics upon the development of fever in these high-risk patients [8].

Over the past two decades, the antibiotic resistance patterns have changed in both gram-positive and gram-negative pathogens that cause infections in febrile neutropenic patients [12]. The emergence of antimicrobial resistance among these pathogens poses new challenges in the management of febrile neutropenic patients. The choice of first-line empiric therapy varies according to local prevalence and bacterial resistance/susceptibility patterns [13].

In our literature review, we found limited numbers of studies addressing bacterial spectrum, isolation sites and susceptibility patterns in febrile neutropenic patients in Saudi Arabia, and our study is the first comprehensive report to address these issues [14, 15].

## Methods

A retrospective study was conducted on the bacterial spectrum, isolation sites and susceptibility patterns of pathogens in adult febrile neutropenic patients hospitalized between May 2009 and May 2013 at the King Fahad Specialist Hospital, a referral hospital providing tertiary care for the Eastern province of Saudi Arabia, with Oncology and Transplant as core competencies. The study was approved by the hospital IRB committee. The patients were included if they met the following inclusion criteria: 1) male and female over the age of 18 years; 2) presence of neutropenia (absolute neutrophil count of < 500 cells/mm^3^ or predicted decrease below 500 cells/mm^3^ during the next 48 h; 3) having a single oral temperature measurement of ≥ 38.3 °C (101 °F) or a temperature of ≥ 38.0 °C (100.4 °F) sustained over 1 h period; 4) having known malignancies; 5) patients with presumed infectious cause of fever were included as high risk. The definition of fever neutropenia was based on the Infectious Diseases Society of America (IDSA) and National Comprehensive Cancer Network (NCCN) clinical guidelines in neutropenic fever in cancer patients. All the data were collected from electronic hospital information system, MedicaPlus. All microbiology reports were as per Clinical and Laboratory Standard Institute (CLSI) guidelines.

## Results

**Figure 1:**
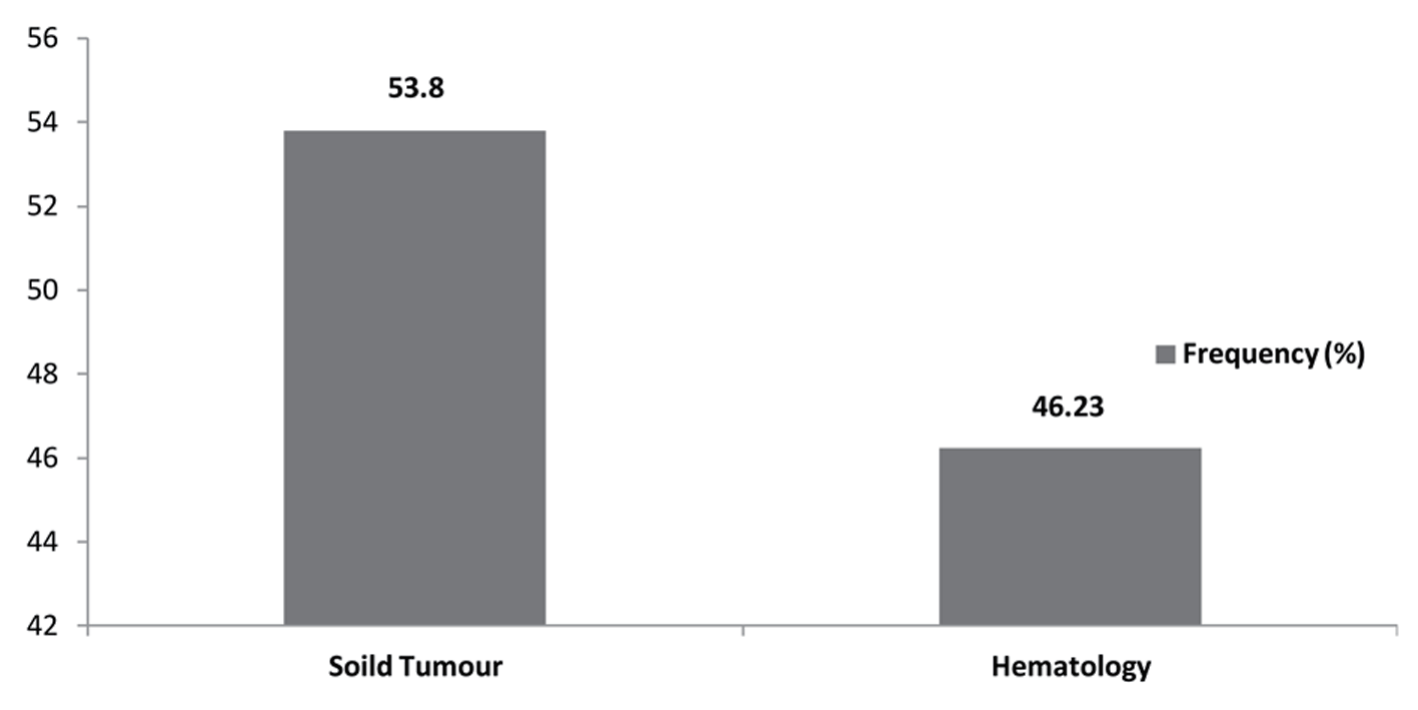
Frequency of type of malignancy.

**Figure 2:**
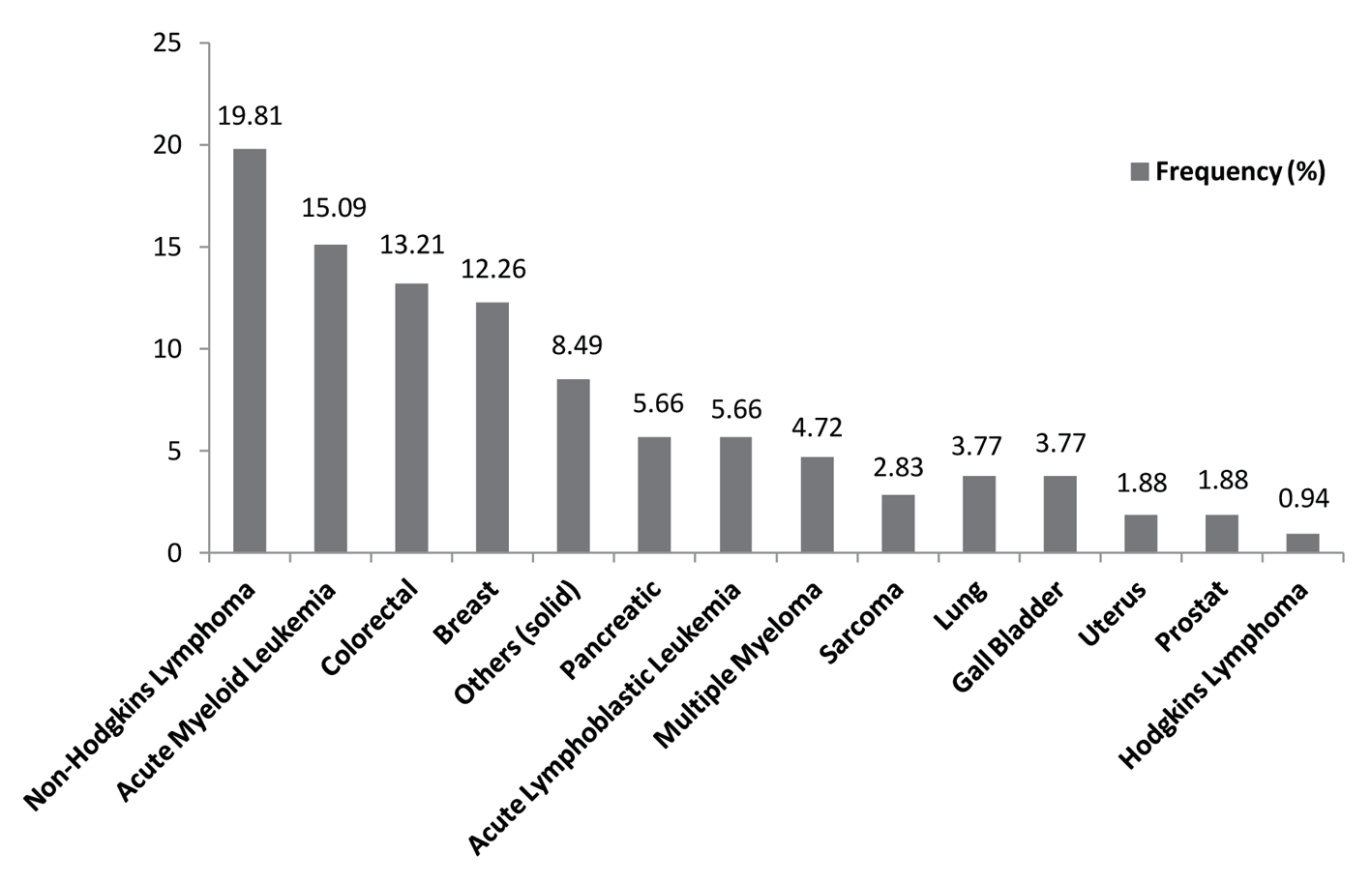
Prevalence of malignancies.

Between May 2009 and May 2013, a total of 106 microbiologically documented infections in febrile neutropenic cancer patients were studied, 53.77% (57/106) of the infections were in patients with solid tumours and 46.23% (49/106) were in patients with hematological malignancies. The distribution of malignancies showed that non-Hodgkin’s lymphoma 19.81% (21/106) was the commonest, followed by acute myeloid leukemia 15.09% (16/106), colorectal cancer 13.21% (14/106), breast cancer 12.26% (13/106) and acute lymphoblastic lymphoma and pancreatic cancer 5.66 (6/106) each (Table 1, Fig. 1, 2).

**Table 1:** Frequency of Malignancy Type

Malignancy type	Number	Frequency (%)
Solid tumors		
Colorectal	14	13.21
Breast	13	12.26
Pancreatic	6	5.66
Gall bladder	4	3.77
Lung	4	3.77
Sarcoma	3	2.83
Prostate	2	1.88
Uterus	2	1.88
Others	9	8.49
Total frequency of solid tumors	57	53.77
Hematology		
Leukemia		
Acute myeloid leukemia	16	15.09
Acute lymphoblastic leukemia	6	5.66
Lyphoma		
Non-Hodgkin’s lymphoma	21	19.81
Hodgkin’s lymphoma	1	0.94
Myeloma		
Multiple myeloma	5	4.72
Total frequency of hematology	49	46.23

### Bacterial etiology, sites of isolation and susceptibility pattern

Overall 138 organisms were isolated. In solid tumors, gram-negative organisms accounted for 71.05% (54/76) of the total infections, while gram-positive organisms accounted for 28.95% (22/76) where as in hematological malignancies, gram-negative organisms accounted for 59.68% (37/62), and gram-positive organisms accounted for 40.32% (25/62) (Table 2).

**Table 2:** Frequency of Gram-Positive and Gram-Negative Pathogens

Malignancy type	Gram-positive	Frequency (%)	Gram-negative	Frequency (%)
Solid tumors	22	28.95	54	71.05
Hematology	25	40.32	37	59.68

**Figure 3:**
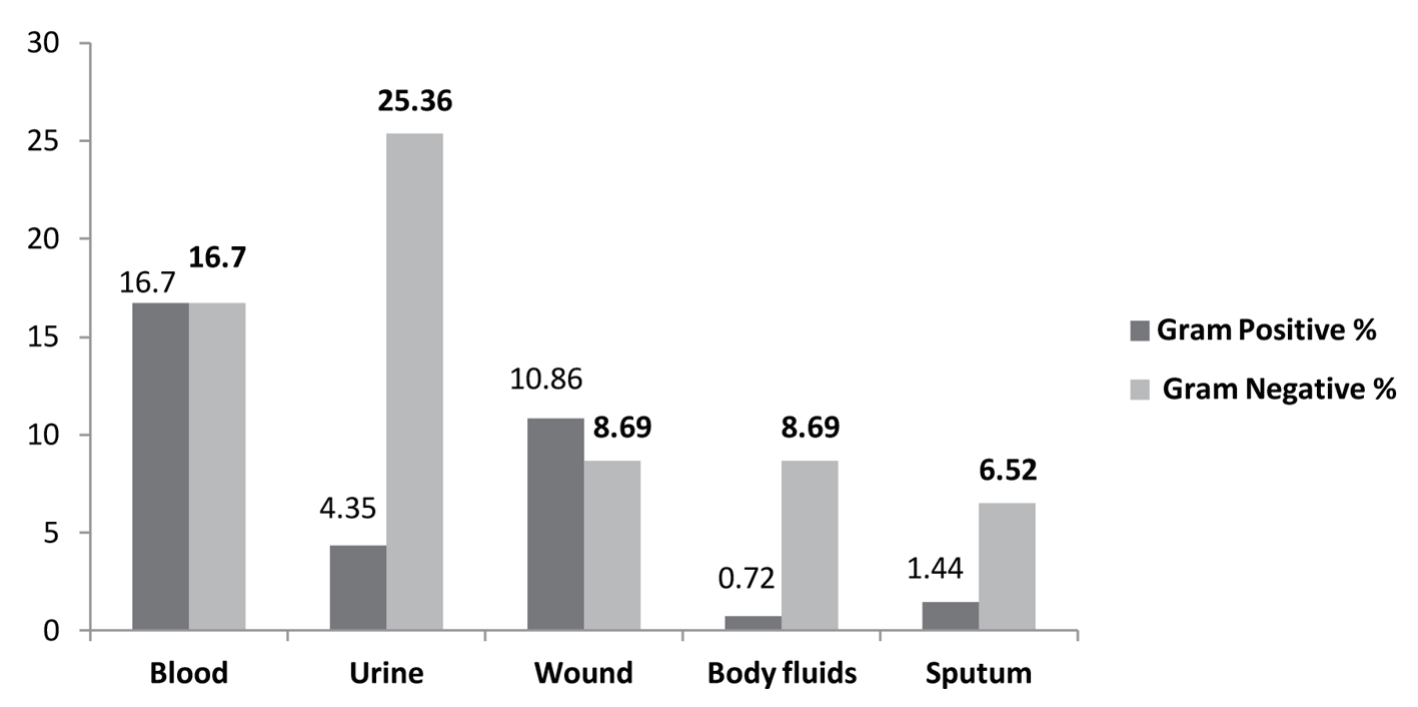
Frequency of pathogens at isolated sites.

The most common source of bacterial isolation sites was blood stream infections 33.33% (46/138) followed by urine 29.71% (41/138), wound 19.56% (27/138), body fluids 9.42% (13/138) and sputum 7.97% (11/138) (Table 3, Fig. 3).

**Table 3:** Frequency of Pathogens at Isolated Sites

Isolation sites	Gram-positive	%	Gram-negative	%
Blood	23	16.7	23	16.7
Urine	6	4.35	35	25.36
Wound	15	10.86	12	8.69
Body fluids	1	0.72	12	8.69
Sputum	2	1.44	9	6.52

Gram-negative organisms attributed to 16.7% (23/138) of blood stream infections and 25.36% (35/138) of urinary tract infections while gram-positive organisms accounted for 16.66% (23/138) of the blood stream infections and 10.86% (15/138) of wound infections (Table 3, Fig. 3).

**Figure 4:**
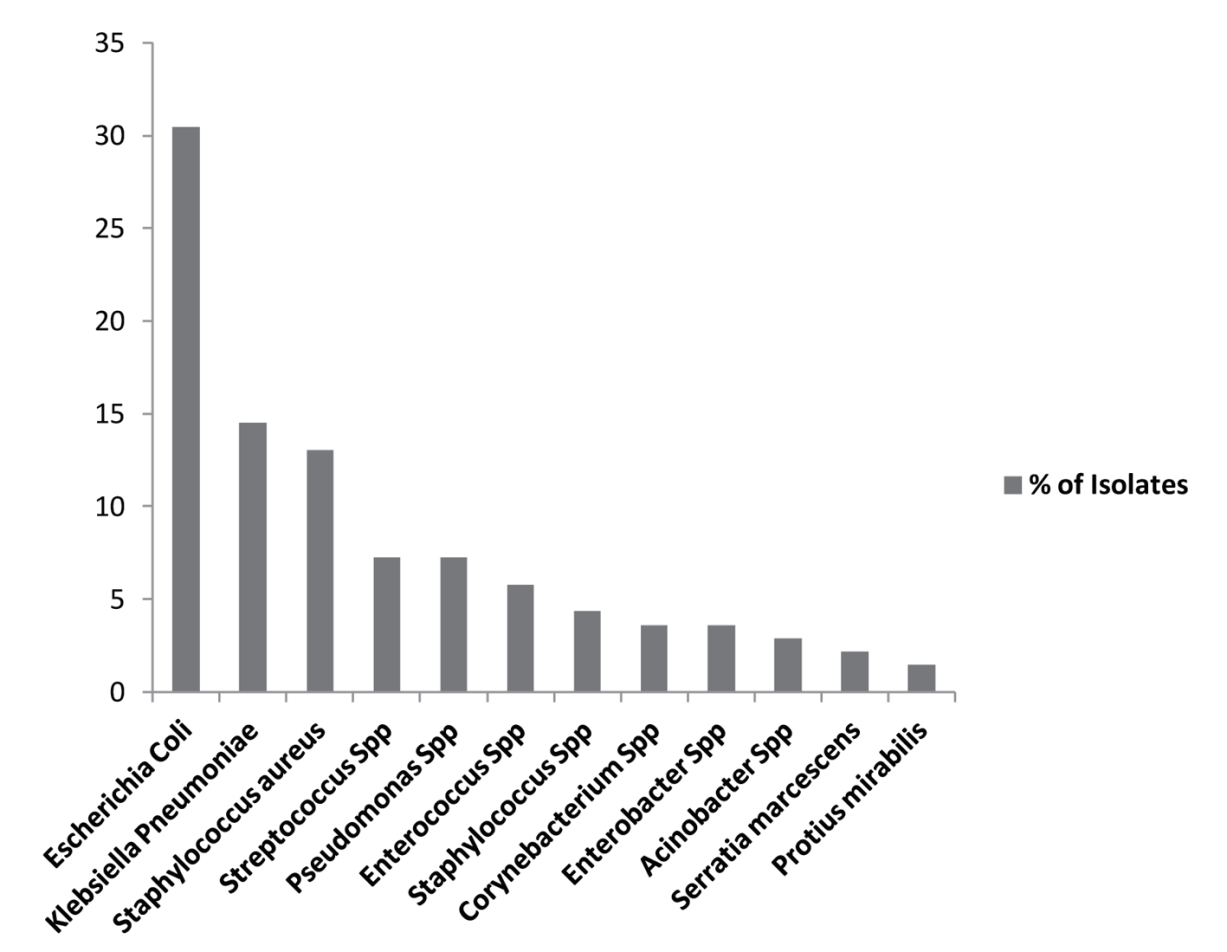
Prevalence of pathogens in febrile neutropenia.

*Escherichia coli* was the most predominant isolate overall accounting for 30.43% (42/138), *Klebsiella pneumonia* accounted for 14.49% (20/138), *Pseudomonas* species 7.25% (10/138), *Streptococcus* species 7.25% (10/138), *Enterococcus* species 5.8% (8/138) and *Staphylococcus aureus* 4.35% (6/138) (Table 4, 5, Fig. 4).

**Table 4:** Frequency of Gram-Positive Bacterial Isolates in Febrile Neutropenia

Gram-positive bacteria	Blood culture	Urine culture	Sputum culture	Wound culture	Body fluids culture	Total (%)
*Staphylococcus aureus*	5	-	1	11	1	18 (13.04%)
*Staphylococcus* spp. (CoNS)	4	1	-	1	-	6 (4.35%)
*Streptococcus* spp.	6	2	1	1	-	10 (7.25%)
*Enterococcus* spp.	4	3	-	1	-	8 (5.80%)
*Corynebacterium* spp.	4	-	-	1	-	5 (3.62%)
Overall frequency						47 (34.06%)

**Table 5:** Frequency of Gram-Negative Isolates in Febrile Neutropenia

Gram-negative bacteria	Blood culture	Urine culture	Sputum culture	Wound culture	Body fluids culture	Total (%)
*Escherichia coli*	9	20	1	5	7	42 (30.43%)
*Klebsiella pneumoniae*	7	6	3	2	2	20 (14.49%)
*Pseudomonas* spp.	1	2	4	3	-	10 (7.25%)
*Enterobacter* spp.	2	2	-	-	1	5 (3.62%)
*Acinobacter* spp.	2	2	-	-	-	4 (2.90%)
*Serratia marcescens*	-	2	-	-	1	3 (2.17%)
*Proteus mirabilis*	-	-	-	2	-	2 (1.45%)
*Aeromonas hydrophylia*	1	-	-	-	-	1 (00.72%)
*Citrobacter freundii*	-	1	-	-	-	1 (0.72%)
*Providencia stuartii*	1	-	-	-	-	1 (0.72%)
*Sphingomonas paucimobilis*	-	-	-	-	1	1 (0.72%)
*Stenotropomonas multipholia*	-	-	1	-	-	1 (0.72%)
Overall frequency						91 (65.94%)

**Figure 5:**
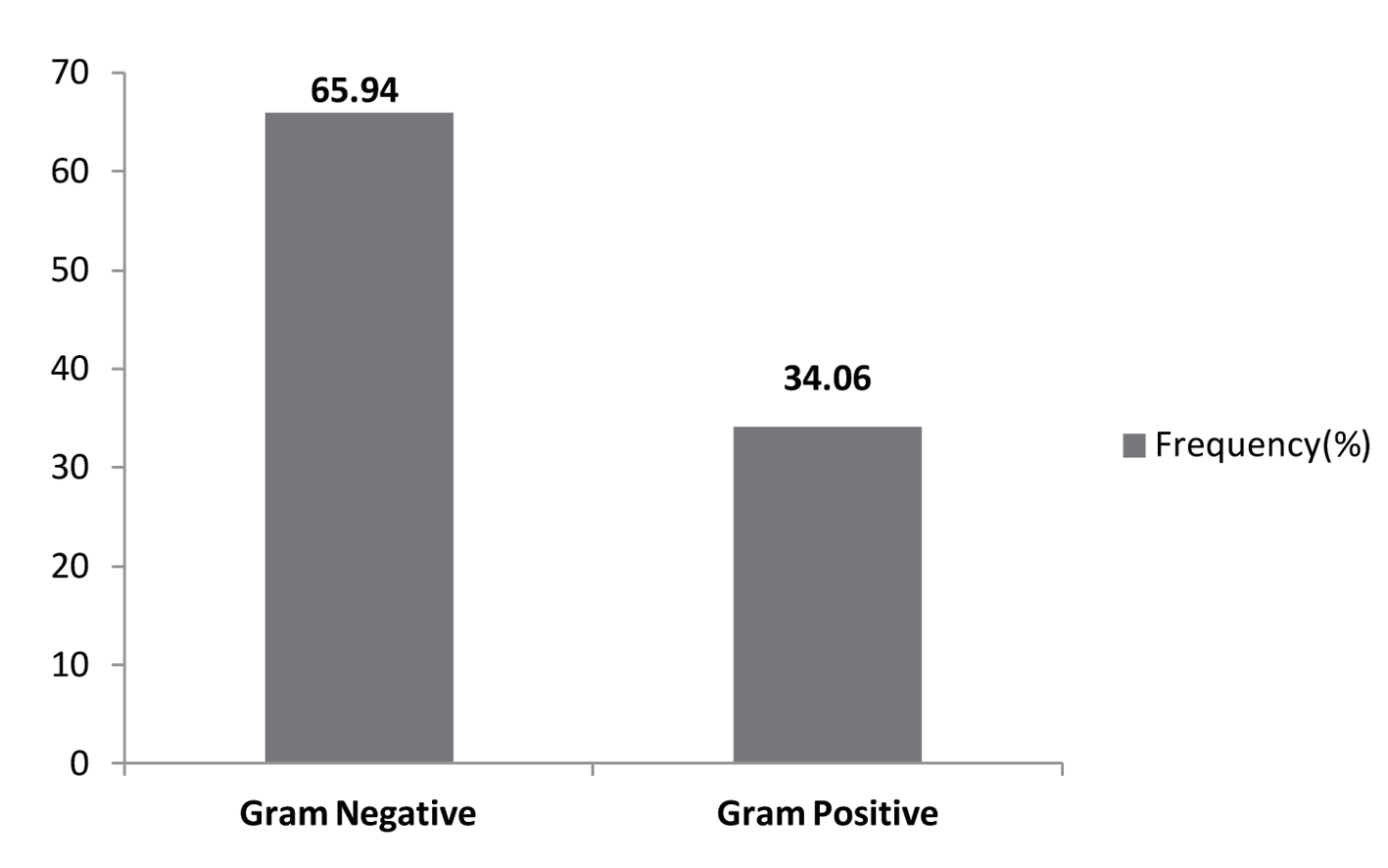
Overall frequencies of bacterial isolates.

In our study, the majority of pathogens were gram-negative organisms accounting for 65.94% (91/138) of total isolates, while gram-positive organisms accounted for 34.06% (47/138) (Table 4, 5, Fig. 5).

We examined the susceptibility patterns of the predominant gram-negative and gram-positive pathogens. Among gram-negative pathogens, *Escherichia coli* showed the following susceptibility patterns: imipenem-cilastatin (99.86%), amikacin (91.6%), piperacillin-tazobactam (83.15%) and ceftriaxone (62.06%); ESBLs rate was 38%. While *Klebsiella pneumonia* showed the following susceptibility patterns: imipenem-cilastatin (98.96%), amikacin (95.24%), ciprofloxacin (75.42%) and ceftriaxone (77.78%); ESBLs rate was 22.22%. The *Pseudomonas aeruginosa* isolates showed the following susceptibility patterns: amikacin (88.92%), ciprofloxacin (82.62%), piperacillin-tazobactam (82.28%) and imipenem-cilastatin (81.16%). *Acinobacter baumanii* showed high resistance to ciprofloxacin (65.5%) and imipenem-cilastatin (56.65%).

For gram-positive organisms, methicillin-resistant *Staphylococcus aureus* (MRSA) rate was (28.62%). *Enterococcus* showed vancomycin-resistant rate in 1.2% of isolates. Table 6 summarizes frequency of susceptibility patterns of most frequently used antimicrobials in the empirical treatment of infections in febrile neutropenic patients at King Fahad Specialist Hospital, Dammam, Saudi Arabia.

**Table 6:** Susceptibility of Gram-Negative and Gram-Positive Isolates

Gram-negative isolates	Isolates	AM	CF	GM	IC	PT	CT	
*Escherichia coli*	42	91.6	47.1	67.36	99.86	83.15	62.06	
*Klebsiella pneumonae*	20	95.24	75.42	82.12	98.96	69.48	77.78	
*Pseudomonas aeugenosa*	10	88.92	82.62	88.52	81.16	82.28	0	
*Enterbacter cloacae*	5	93.52	82.36	86.92	100	66.9	60.76	
*Acinobacter baumanii*	4	67.32	34.5	52.86	43.74	28.14	2.45	
*Serratia marscences*	3	100	95	97.34	100	93.2	88.6	
*Proteus mirabilis*	2	95.98	68.36	77.34	94.38	93.52	85.5	
Gram-positive isolates	Isolates	CF	GM	VM	LF	LZ	CT	OC
*Staphylococcus aureus*	18	72.05	89.38	100	74.36	100	0	71.38
*Streptococcus pneumoniae*	10	100	100	100	100	100	96.9	69.1
*Enterococcus* spp.	8	51.45	35.65	98.82	63.04	96.58	0	0
Coagulase-negative *Staphylococcus*	6	38.7	53.1	99.76	38.5	99.22	0	12.88

AM: amikacin; CF: ciprofloxacin; GM: gentamycin; IC: imipenem-cilastatin; PT: piperacillin-tazobactam; CT: ceftriaxone; VM: vancomycin; LF: levofloxacin; LZ: linezolid; OC: oxacillin.

## Discussion

Infection is a most common complication of chemotherapy, and it causes morbidity and mortality in neutropenic cancer patients. Fever may be the only indication of infection in febrile neutropenia. Bacterial infections are common in this population. In order to treat effectively, the knowledge of likely pathogens and local bacterial spectrum is very important [16-19].

### Trend in shifting of bacterial etiology

In the past two decades, there was a shift in the etiology of bacterial infections in febrile neutropenic patients towards gram-positive organisms accounting for as much as 70% in various reports [7]. The cause of this change in etiology is unclear. This change might be due to long-term use of indwelling catheters, radiation therapy, widespread use of flouroquinolones and aggressive chemotherapy leading to mucosal damage and increasing the risk of infection [6, 7, 10]. The nature of chemotherapy used also influences bacterial etiology. The regional climatic or environmental conditions also influence changes in bacterial spectrum. *Pseudomonas aerugenosa* are more predominant in warmer climates [9].

Recently, the bacterial etiology has changed again from gram-positive to gram-negative organisms. Many studies from different parts of the world showed this change of trend [5, 11, 18, 20-22] and only one small study from Saudi Arabia looked at the pattern of febrile neutropenia presentations in solid tumors [14, 15].

In our study of the etiology of bacterial infections in febrile neutropenic patients, gram-negative pathogens (65.94%) accounted for almost two-thirds of bacterial infections whereas gram-positive organisms were identified in 34.06%. Our findings are similar to other studies in our geographical region, pointing to the importance of covering gram-negative organisms empirically according to the most likely pathogens, and local sensitivity data in this high-risk group of patients [16, 18].

Our data provide a greater emphasis on the importance of local data in managing patients as described in the literature and a combination of an antipseudomonal beta-lactam agent and an aminogylcoside empirical regimen was adapted in our hospital according to our data; vancomycin was added for patients with MRSA risk factors.

### Site of bacterial isolation

There are many studies showing the importance of geographical location in bacterial isolation sites [2, 18, 21, 23]. In the published literature, the most common bacterial isolation sites in patients with febrile neutropenia were blood stream, urine, wound, respiratory tract, gastrointestinal tract and skin and soft tissues [7, 22, 24].

Our study showed similar trends of site of isolation as discussed in the above studies. The most predominant isolation sites were blood stream 33.32%, urine 29.71%, wounds 19.55%, body fluids 7.96%, and from sputum 7.96%.

### Susceptibility patterns

Extended-spectrum β-lactamase (ESBL)-producing bacteria have become a serious problem in many different geographical regions. Carbapenems are the corner stone of treatment for these organisms, and because of its increase usage for empirical therapy, the risk of selecting resistant organisms is increasing (carbapenem-producing organisms). Many centers are also reporting increased rates of multidrug-resistant gram-negative bacteria such as *Acinobacter and Pseudomonas aerugenosa*, which makes treatment of these infections more difficult [25]. Studies also reported the increased rates of gram-positive resistant pathogens in many centers, especially MRSA and VRE [26].

In our study we found high rates of ESBLs among *Escherichia coli* (38%) and *Klebsiella pneumonia* (22.22%) and increased imipenem-cilastatin resistance among *Pseudomonas aerugenosa* (18.84%); MRSA rate was 28.72%. This higher than reported rate of ESBLs in our hospital compared to the published rates in the region is probably due to the nature of our high-risk cancer patients, recurrent hospitalizations, and prior use of antimicrobials and the increased use of antibiotics for prophylaxis especially flouroquinolones [27].

### Conclusions

In conclusion, our study shows similar results with international studies in overall prevalence of gram-negative organisms and their isolation sites but different trends in bacterial etiology and susceptibility patterns. Local data for bacteria causing infections in febrile neutropenic patients are needed to help in selecting appropriate empirical antimicrobial therapy.
